# *Zingiber officinale*-Derived Extracellular Vesicles Attenuate Bleomycin-Induced Pulmonary Fibrosis Trough Antioxidant, Anti-Inflammatory and Protease Activity in a Mouse Model

**DOI:** 10.3390/cells12141852

**Published:** 2023-07-14

**Authors:** Alma Aurora Ramírez-Hernández, Edilburga Reyes-Jiménez, Juan Manuel Velázquez-Enríquez, Jovito Cesar Santos-Álvarez, Adriana Soto-Guzmán, Luis Castro-Sánchez, Gabriela Tapia-Pastrana, Honorio Torres-Aguilar, Verónica Rocío Vásquez-Garzón, Rafael Baltiérrez-Hoyos

**Affiliations:** 1 Laboratorio de Fibrosis y Cáncer, Facultad de Medicina y Cirugía, Universidad Autónoma Benito Juárez de Oaxaca, Oaxaca de Juárez 68120, Mexico; aramih_09@cecad-uabjo.mx (A.A.R.-H.); edilreyesjimnez@cecad-uabjo.mx (E.R.-J.); juanmanuelvela_enriquez@cecad-uabjo.mx (J.M.V.-E.); jovitocesarsa@cecad-uabjo.mx (J.C.S.-Á.); vrvasquezga@conacyt.mx (V.R.V.-G.); 2 Departamento de Medicina y Ciencias de la Salud, Universidad de Sonora, Hermosillo 83000, Mexico; adriana.soto@unison.mx; 3 CONAHCYT-Centro Universitario de Investigaciones Biomédicas, Universidad de Colima, Colima 28045, Mexico; luis_castro@ucol.mx; 4 Laboratorio en Investigación Biomédica, Hospital Regional de Alta Especialidad de Oaxaca, San Bartolo Coyotepec, Oaxaca de Juárez 71256, Mexico; gtapia@hraeoaxaca.gob.mx; 5 Facultad de Ciencias Químicas, Universidad Autónoma Benito Juárez de Oaxaca, Av. Universidad S/N, Cinco Señores, Oaxaca de Juárez 68120, Mexico; qbhonorio@hotmail.com; 6 CONAHCYT-Facultad de Medicina y Cirugía, Universidad Autónoma Benito Juárez de Oaxaca, Oaxaca de Juárez 68120, Mexico

**Keywords:** protease activity, SOD, planta extracellular vesicles, antioxidants, idiopathic pulmonary fibrosis, lung inflammation

## Abstract

Idiopathic pulmonary fibrosis (IPF) is the most frequent and severe idiopathic interstitial pneumonia. It is a chronic and progressive disease with a poor prognosis and is a major cause of morbidity and mortality. This disease has no cure; therefore, there is a clinical need to search for alternative treatments with greater efficacy. In this study, we aimed to evaluate the effect of extracellular vesicles (EVs) from *Zingiber officinale* (EVZO) in a murine model of bleomycin (BLM)-induced IPF administered through an osmotic minipump. EVZO had an average size of 373 nm and a spherical morphology, as identified by scanning electron microscopy. Label-free proteomic analysis of EVZOs was performed by liquid chromatography coupled to mass spectrometry, and 20 proteins were identified. In addition, we demonstrated the protease activity of EVZO by gelatin-degrading zymography assay and the superoxide dismutase (SOD) activity of EVZO by an enzymatic assay. In the BLM-induced IPF mouse model, nasal administration of 50 μg of EVZO induced recovery of alveolar space size and decreased cellular infiltrate, collagen deposition, and expression of α-SMA-positive cells. Additionally, EVZO inhibited inflammatory markers such as iNOS and COX-2, lipid peroxidation, and apoptotic cells. These results show that EVZO may represent a novel natural delivery mechanism to treat IPF.

## 1. Introduction

The American Thoracic Society, the European Respiratory Society, and the American College of Chest Physicians define idiopathic pulmonary fibrosis (IPF) as a chronic age-related, progressive disease of unknown etiology that has a poor prognosis with a median survival of three to five years after diagnosis and is more common in males and elderly adults with an incidence of 0.09 to 1.30 per 10,000 persons and a prevalence of 0.33 to 4.51 per 10,000 persons globally [1,2]. IPF is characterized by excessive extracellular matrix (ECM) deposition, epithelial/endothelial and/or alveolar basement membrane destruction, and proliferation of various cell populations, including fibroblasts/myofibroblasts responsible for synthesizing ECM proteins, causing dysfunction in gas exchange, respiratory failure, and eventually death [3].

Several therapeutic approaches have been focused on treating IPF; however, despite constant progress in the search for new therapies, it has not been possible to improve the survival of patients. Two antifibrotic therapies have been approved by the Food and Drug Administration (FDA): pirfenidone and nintedanib [4]. Currently, monotherapy with these drugs has shown significant progress; however, a cure has not been found, and these drugs have some disadvantages due to side effects such as nausea, skin rashes, diarrhea, dyspepsia, and dizziness [5,6,7].

Therefore, several studies have focused mainly on natural products [8,9], which are considered a reservoir of bioactive components with therapeutic properties released through small extracellular vesicles (EVs) [10]. EVs are heterogeneous spherical nanostructures formed by a lipid bilayer that carries various bioactive cargoes, such as proteins, nucleic acids, and metabolites. They have an average size of 50–150 nm and can vary according to their origin [11,12,13]. Plant EVs were first observed in 1967 before mammalian EVs, and they are similar to mammalian exosomes and differ in their metabolite content. Plant EVs are being studied with new therapeutic approaches; due to their nature, they are not detected by the immune system and present a higher bioavailability compared to their homologs from mesenchymal stem cells [14,15].

*Zingiber officinale* (ginger) has acquired great importance in health and the treatment of various diseases. Previous reports have shown that the total extract of ginger contains metabolites and EVs that possess anti-inflammatory and antioxidant properties [16,17,18,19,20,21]. Some studies have shown that EVs from carrot, grape, grapefruit, lemon, and ginger, among others, have anti-inflammatory properties in models of colitis and liver damage [13,22,23,24] that have been administered intragastrically and orally [13,25,26] To our knowledge, the effects generated by ginger EVs have not been studied in pulmonary pathologies. In this study, we used extracellular vesicles from *Zingiber officinale* (EVZO) to evaluate for the first time its effect in a bleomycin (BLM)-induced IPF model. Taken together, the present study demonstrated for the first time that nasal administration of EVZO in IPF mice decreased histopathological damage leading to a recovery of lung architecture, thus providing a breakthrough in discovering new therapeutic alternatives for IPF.

## 2. Materials and Methods

### 2.1. Plant Material and EVZO Isolation

**Figure 1:**
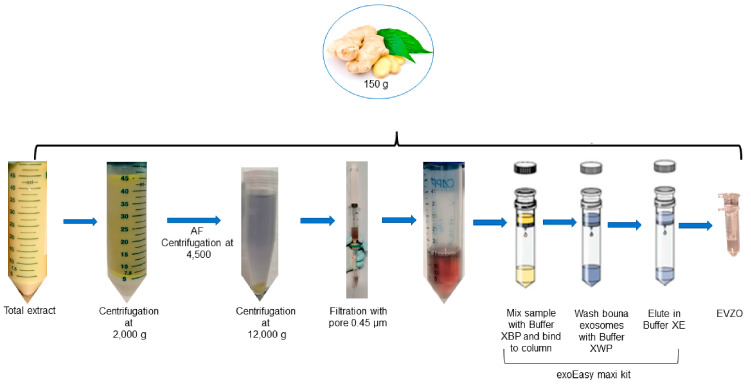
Scheme for obtaining EVs derived from ginger. Ginger extract was subjected to differential centrifugation (2000× *g*, 4500× *g*, and 15,000× *g*) to remove fibers and cell detritus, and the final supernatant was used to isolate EVs using the exoEasy Maxi kit. AF = aqueous phase; EVZO = extracellular vesicles of *Zingiber officinale*.

Fresh ginger rhizomes (150 g) were used for each assay, which were obtained from the local market, washed with distilled water, peeled, and cut into small pieces so that the juice could be extracted with a 500 W extractor for 2 min. The total extract of fresh *Zingiber officinale* (TEZO) obtained was sequentially centrifuged (Centrifuge Eppendorf 5804 R, Hamburg, Germany) at 2000× *g* for 15 min, 4500× *g* for 30 min, and 12,000× *g* for 30 min at 4 °C to remove fibers and cell debris, respectively. The supernatant obtained was filtered through a 0.45 µM filter (Whatman, Buckinghamshine, UK), and isolation of EVs was performed with the exoEasy Maxi kit (76064, Qiagen, Hilden, Germany) according to the manufacturer’s instructions [27]. Subsequently, they were ultraconcentrated at 12,000× *g* for 15 min at 20 °C using Vivaspin 500 100,000 MWCO PES tubes (VS0141,Sartorius, Stonehouse, UK) and stored at 4 °C (Figure 1).

### 2.2. Size Determination by Dynamic Light Scattering

The size distribution of EVZO was determined by dynamic light scattering (DLS) on a Malvern Zetasizer NanoZS instrument (Malvern Instruments, Malvern, UK). In brief, 100 µL of EVZO was mixed with 1 µL of 0.5 M EDTA pH 8.0 in sterile 1X PBS pH 7.4 and diluted in 20 mM HEPES (Sigma-Aldrich, St. Louis, MO, USA) for measurement with a resolution of 0.5 nm and a sensitivity of 0.1 ppm at 25 °C. The size data of the EVs were analyzed and plotted with Origin (OEM version) 2016 software.

### 2.3. Scanning Electron Microscopy (SEM)

The morphological evaluation of the EVZO was performed using a microscope (Field Emission Scanning Electron Microscope Model JSM 7800 F; JEOL Ltd., Akishima-shi, Japan). Ten microliters of EVs were deposited on a formvar/carbon-coated copper SEM grid (300 Mesh), and after 20 s, the excess was removed with filter paper. Samples were stained using 10 µL of 2% phosphotungstic acid solution for 15 s. The grid was vacuum-dried for 24 h before observations (20 kV).

### 2.4. Label-Free Proteomic Analysis

#### 2.4.1. Chemicals

DL-dithiothreitol (DTT), iodoacetamide (IAA), formic acid (FA), acetonitrile (ACN), and methanol were purchased from Sigma (St. Louis, MO, USA). Trypsin from the bovine pancreas was purchased from Promega (Madison, WI, USA). Ultrapure water was prepared using a Millipore purification system (Billerica, MA, USA).

#### 2.4.2. Sample Preparation

First, cold acetone (Hycel, 900, Guadalajara, Mexico) precipitated the total protein per sample from the protein solution. Protein pellets were dissolved in 2 M urea aqueous solution and denatured with 10 mM DTT incubated at 56 °C for 1 h, followed by alkylation with 50 mM IAA. The solution was incubated for 60 min at room temperature (RT) in the dark. Then, 500 mM ammonium bicarbonate was added to the solution to a final concentration of 50 mM ammonium bicarbonate with a pH of 7.8. Then, Promega Trypsin was added into the protein solution for digestion at 37 °C for 15 h. The generated peptides were purified with a C18 SPE column (Thermo Scientific, Waltham, MA, USA) to remove the salt. Finally, the extracted peptides were lyophilized to near dryness. The peptides were resuspended in 20 μL of 0.1% formic acid before LC-MS/MS analysis.

#### 2.4.3. Nano LC-MS/MS Analysis

Nano LC-MS/MS analyses were performed using an Ultimate 3000 nano UHPLC system coupled with a Q Exactive HF mass spectrometer (Thermo Fisher Scientific, Waltham, MA, USA) with an ESI nanospray source. One microgram of sample from collected fractions was loaded onto a trapping column (PepMap C18, 100 Å, 100 μm × 20 cm, 5 μm) and an analytical column (PepMap C18, 100 Å, 75 μm × 50 cm, 2 μm). The mobile phases were (A) 0.1% formic acid in water and (B) 0.1% formic acid in acetonitrile. A linear gradient was applied from 2% to 8% buffer B for 5 min, from 8% to 20% buffer B for 60 min, from 20% to 40% buffer B for 33 min, and then from 40% to 90% buffer B for 4 min at a 250 nL/min flow rate.

#### 2.4.4. Mass Spectrometry

The full scan was performed between 300 and 1650 *m*/*z* at a resolution of 60,000 at 200 *m*/*z*, and the automatic gain control target for the full scan was set to 3.0 × 106. The MS/MS scan was operated in Top 20 mode using the following settings: resolution 15,000 at 200 *m*/*z*; automatic gain control target 1.0 × 10^5^; maximum injection time of 19 ms; normalized collision energy at 28%; isolation window of 1.4 Th; charge state exclusion: unassigned, 1, >6; dynamic exclusion 30 s.

#### 2.4.5. Data Analysis

The raw MS files were analyzed and searched against the *Zingiber officinale* protein database based on the species of the samples using MaxQuant (1.6.1.14) (Max Planck Institute, Martinsried, Germany). The parameters were set as follows: the protein modifications were carbamidomethylation (C) (fixed) and oxidation (M) (variable); the enzyme specificity was set to trypsin; the maximum missed cleavage was set to 2; the precursor ion mass tolerance was set to 10 ppm; and the MS/MS tolerance was 0.5 Da.

#### 2.4.6. Bioinformatics Analysis

A Gene Ontology (GO) annotation analysis of the identified proteins was performed using the search system (https://www.uniprot.org (accessed on 28 February 2022)) to retrieve the GO terms assigned to each protein as molecular function and biological process.

#### 2.4.7. Zymography

The protease activity of the TEZO and EVZO proteins was evaluated in a zymogram; TE from papaya (TEPA) and EVs from papaya (EVPA) were used as positive controls. Samples were loaded on a 10% acrylamide (1610156, BIO-RAD, CA, USA) gel containing 1% gelatin. Following electrophoresis, the gel was washed in 2.5% Triton X-100 for 30 min, followed by incubation in reaction buffer (Tris 0.050 M/CaCl_2_ 10 Mm/NaCl 150 Mm, NaN3 0.02%) for 24 h at 37 °C. The gel was revealed with Coomassie Brilliant Blue (CAS 6104-58-1, Thermo Scientific, Rockford, IL, USA) solution, and the positive areas of protease activity were observed as a transparent band on the blue background.

#### 2.4.8. EVZO SOD Enzyme Activity

To evaluate the superoxide dismutase (SOD) activity of EVZO, identified in the proteomic analysis, we performed an enzymatic assay using a kit (Cayman Chemicals, 706002, MI, USA). The EVZO sample and the standard solution were used for the assay. Once the working reagents were prepared, we added the Radical Detector reagent to the 96-well plate, followed by the inhibitor and the sample. Finally, xanthine oxidase was immediately mixed for a few seconds and incubated with agitation for 30 min at RT. The absorbance was measured at 450 nm in a Multiskan FC spectrophotometer (Thermo Scientific). Once the reading values were obtained, the calibration curve was performed, and the SOD activity value of EVZO was obtained according to the curve in U/mL.

### 2.5. Animals

Male CD1 mice aged 8 to 10 weeks, weighing 38 to 46 g were maintained in 12 h light–dark cycles at 23 °C in a pyrogen-free environment with free access to food and water. The Ethics Committee of the Universidad Autónoma Benito Juárez de Oaxaca approved the experimental protocol with registration number 0047-CEI-2022.

#### 2.5.1. Experimental Design

**Figure 2:**
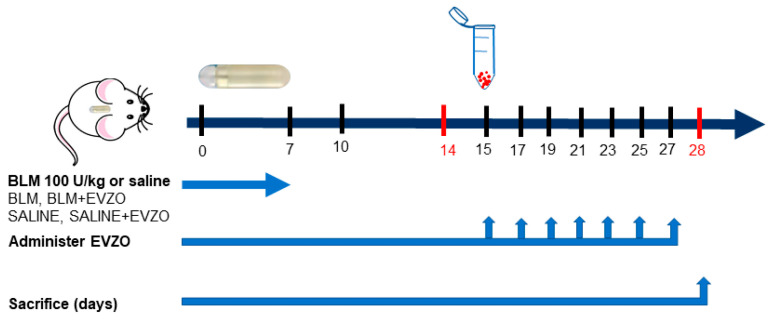
Experimental model. Administration of BLM by osmotic minipumps. Four experimental groups were established, and each group consisted of four CD1 mice, two control groups (saline and saline plus EVZO from day 15), and two BLM groups (BLM 100 U/kg and BLM plus 50 µg EVZO from day 15) every other day. All animals were euthanized on day 28.

The model used was the one proposed by Lee [28], where the administration of BLM is through osmotic minipumps. This route of administration allows a constant and systemic delivery of the drug, and reproduces the distinctive marks of IPF compared to the classical method [28,29]. Mice were randomly divided into four groups: control group 1 (CT) without treatment, control group 2 with EVZO treatment (CT+EVZO), group 3 treated with BLM, and group 4 treated with BLM+EVZO, a BLM dose of 100 U/kg [28] (Bleocel, Celon Labs, Telangana State, India) administered through osmotic minipumps (ALZET 1007D; DURECT, Cupertino, CA, USA) implanted under loose skin posterior to the scapular area under isoflurane anesthesia. On day 15 after BLM administration, treatments were initiated with 50 µg [23] (concerning protein concentration) of EVZO administered nasally to the CT+EVZO and BLM+EVZO groups. Treatments were given on alternate days for a total of 7 administrations. Finally, the mice were sacrificed on day 28, and the lungs were recovered and embedded in paraffin for further analysis (Figure 2).

#### 2.5.2. Histological Analysis

Lungs were fixed with 4% formaldehyde and embedded in paraffin. Slices of 5 μm were taken and collected on gelatinized slides for hematoxylin-eosin (H&E) staining to analyze the general morphology of the tissue and for Masson’s trichrome and Sirius red staining to evaluate collagen deposits. For immunohistochemical analysis, 3 μm slices were taken (Leica, model RM 2125 RTS, Wetzlar, Germany).

For H&E staining, the samples were deparaffinized for 30 min at 56 °C, removed in previously heated xylol for 30 min, the tissues were hydrated in xylol 2, xylol/alcohol, and a series of alcohols in decreasing concentrations, and finally in water. Subsequently, the tissues were incubated in Harrys Hematoxylin (Hycel, 738, Guadalaja, Mexico) for 5 min, washed, and set in acid ethanol, followed by an ammoniacal solution, then a yellowish eosin (Hycel, 688, Guadalajara, Mexico) for 10 min. Finally, they were dehydrated and mounted with synthetic resin for microscopic analysis (Primo Star, CARL ZEISS) at 20× magnification [30].

For Masson’s trichrome (MT) staining (HT15-1KT, Sigma-Aldrich, Germany) according to the manufacturer’s instructions with some modifications, tissue sections were deparaffinized, hydrated, and incubated in Bouin’s solution for 1 h at 56 °C and washed, incubated in Weigert’s ferric hematoxylin (1159730002, Merck, Darmstadt, Germany) for 20 min, washed, had Biebrich acid-scarlet fuchsin solution added for 15 min, continuing with a solution of phosphotungstic acid and phosphomolybdic acid (1:2 v/v for 20 min, and finally, aniline blue for 1 h, acetic acid, deionized water, 96% ethanol, xylol, and finally mounted in synthetic resin for an optical microscope (Primo Star, CARL ZEISS) at 20× magnification.

For Sirius Red staining, the sections were hydrated and stained with Weigert’s ferric hematoxylin (1159730002, Merck, Darmstadt, Germany); the excess was removed and washed with water for 10 min, immersed in picro Sirius red colorant (ab150681, Abcam, Cambridge, UK) for 1 h at RT, and the excess was removed and rinsed with 0.5% acidified water, before immediate immersion in 96% alcohol followed by xylol, and was mounted with resin and observed under the microscope. Thirty fields of 4 mice from each group in the stained sections were analyzed, viewed using an optical microscope (Primo Star, CARL ZEISS, Stuttgart, Germany) at 20× magnification, and images were analyzed using ImageJ 1.53t software (U. S. National Institutes of Health, Bethesda, MD, USA).

#### 2.5.3. Immunohistochemistry

Histological sections of 3 μm were cut, deparaffinized, and incubated in citrate buffer at pH 6.0 in a pressure cooker, permeabilized with phosphate-buffered solution (PBS) Triton 0.1%, blocked endogenous peroxidase with 6% peroxide in methanol for 30 min at RT, followed by blockade of nonspecific sites with 3% bovine serum albumin (BSA) in 1X PBS and incubation for 1 h at RT. Sections were incubated with the anti-rabbit primary antibodies listed in Table 1, prepared in 1% BSA overnight at 4 °C. Then, the tissues were washed with PBS and incubated with the anti-rabbit polyclonal secondary antibody for 1 h at 37 °C. The signal was detected with a diaminobenzidine kit (DAB SK-4100, Burlingame, CA, USA), counterstained with Harris hematoxylin, and observed under an optical microscope (Primo Star, CARL ZEISS) at 20× magnification.

#### 2.5.4. Statistical Analysis

Experimental results were quantified with ImageJ 1.53t software and one-way ANOVA in GraphPad 8 Prisma software (GraphPad, San Diego, CA, USA), followed by Tukey’s multiple comparisons tests. All data obtained were expressed as the mean ± standard deviation (SD) of four experimental replicates.

## 3. Results

### 3.1. Isolation and Characterization of EVZO

**Figure 3:**
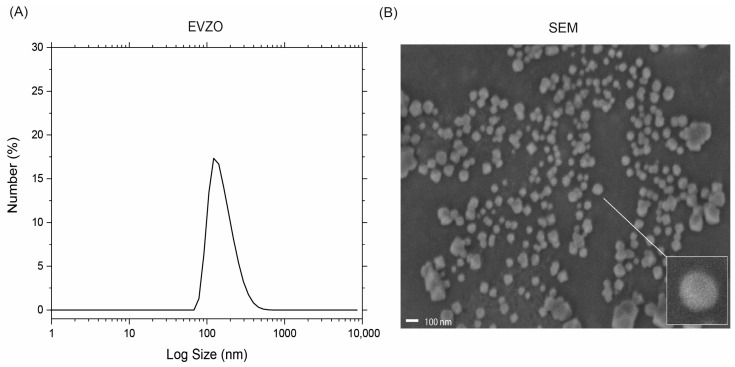
Characterization of EVs derived from ginger. (**A**) The size of EVZO was measured using a Malvern Zetasizer NanoSZ. (**B**) The morphology of EVZO was identified with SEM. The scale bar indicates 100 nm. Results are the mean of two independent samples. SEM = scanning electron microscope; EVZO = extracellular vesicles *Zingiber officinale*; nm = nanometers.

EVZO was isolated and purified from ginger extract using the exoEasy Maxi kit. DLS analysis was performed to confirm the isolation of EVs, and the results showed an average size of 327 ± 52.22 nm (Figure 3A). SEM analysis showed that EVZO had a spherical morphology (Figure 3B). Taken together, these data suggest that we successfully isolated EVZO and are in accordance with those previously reported [13].

### 3.2. Proteomic Analysis of EVZO

A label-free shotgun proteomic analysis was performed to identify the protein contained in EVZO, showing a total of 20 proteins (Table 2).

### 3.3. GO Analysis of Proteins Identified in EVZO

**Figure 4:**
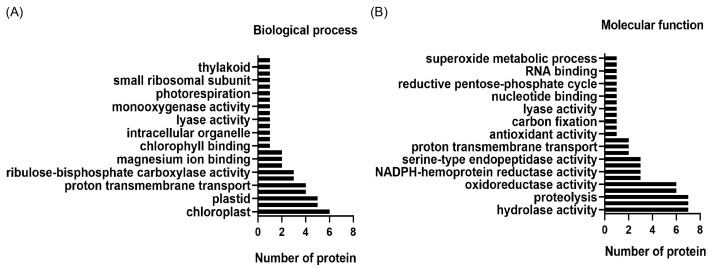
GO analysis shows proteins involved in molecular functions and physiological processes. A GO term plot of molecular function (**A**) and biological processes (**B**) obtained from UniProt is shown.

The identified proteins were included in a GO analysis in order to understand their molecular function and involvement in biological processes through the site https://www.uniprot.org (accessed on 28 February 2022. The analysis revealed that these proteins are involved in molecular functions such as hydrolase activity, proteolysis, oxidoreductase activity, antioxidant activity, and superoxide metabolic processes (Figure 4A) and participate in the biological functions of transmembrane proton transport, intracellular organelle, chlorophyll binding, and photorespiration (Figure 4B).

### 3.4. Effect of EVZO on Bleomycin-Generated Histological Alterations in the Murine Model

**Figure 5:**
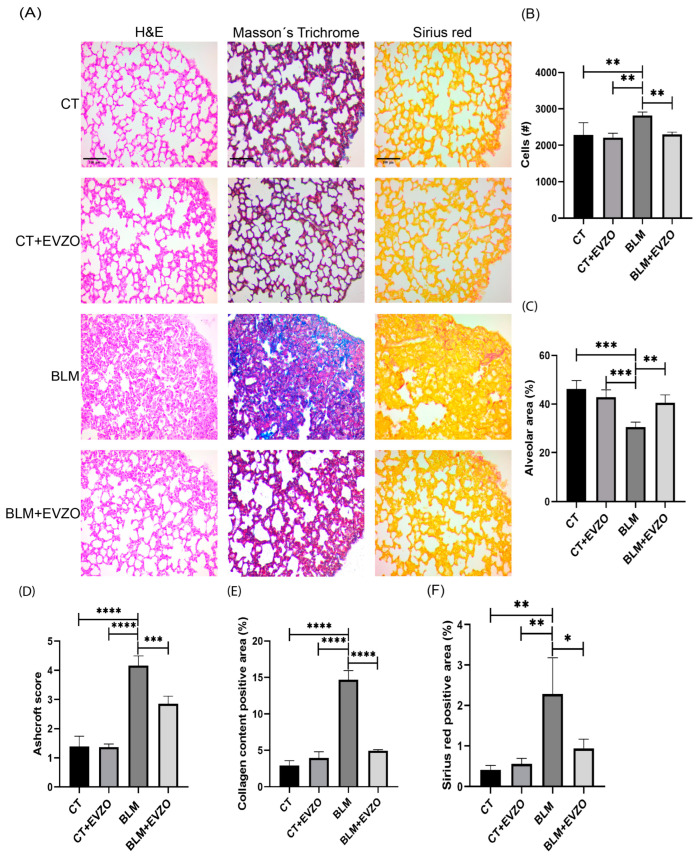
Modulation of fibrosis with EVZO treatment. (**A**) Representative images were taken at 20× magnification of tissue sections were stained with hematoxylin-eosin (H&E), Masson’s trichrome, and Sirius red. (**B**) Quantification of the number (#) of cells, (**C**) measurement of the size of alveolar spaces, (**D**) score of fibrosis, (**E**) quantification of positive areas in Masson’s trichrome staining, and (**F**) quantification of positive areas of Sirius Red. Data are expressed as the mean ± SD (*n* = 4). Data are analyzed by one-way ANOVA followed by Tukey´s t-test for comparison between groups. Quantified with ImageJ 1.53t software and analyzed in GraphPad Prism. * *p* < 0.05, ** *p* < 0.01, *** *p* < 0.001, **** *p* < 0.0001. BLM: bleomycin, CT: control, EVZO: extracellular vesicles from *Zingiber officinale*.

Continuous administration of BLM via osmotic minipumps is the commonly used fibrosis model to reproduce hallmarks of interstitial lung disease, such as changes in lung architecture, fibroblast activation, differentiation to myofibroblasts, and collagen deposition [28]. To evaluate the histological alterations generated by BLM and the effects of EVZO, H&E staining was performed. The analysis showed that the CT and CT+EVZO groups maintained normal parenchyma and alveolar spaces. In the BLM group, a significant increase in cellular infiltrate, thickening of the septum, and a reduction in the size of the alveolar spaces were observed. In contrast, the BLM+EVZO group showed a significant recovery of the size of the alveolar spaces and a decrease in the thickness of the septum and cellular infiltrate (Figure 5A–C).

In addition, collagen deposits and the fibrosis score were evaluated by Masson’s trichrome staining, and the fibrosis score was assessed according to the Ashcroft score. The CT and CT+EVZO groups presented a basal Ashcroft score, whereas the BLM group showed the highest score. The BLM+EVZO group showed a significantly reduced score compared to the BLM group (Figure 5A,D). Subsequently, when quantifying collagen deposits on Masson’s trichrome and Sirius red staining, we obtained a similar behavior showing a significant decrease in collagen deposits with EVZO treatment compared to the BLM group. The CT and CT+EVZO groups showed basal collagen expression in both stainings (Figure 5A,E,F). With these results, we suggest that nasal administration of EVZO decreased cellular infiltrate, collagen deposition, and fibrosis score, consequently increasing the size of the alveolar spaces.

### 3.5. Proteolytic Activity of EVZO in Gel Polymerized Gelatin Gel

**Figure 6:**
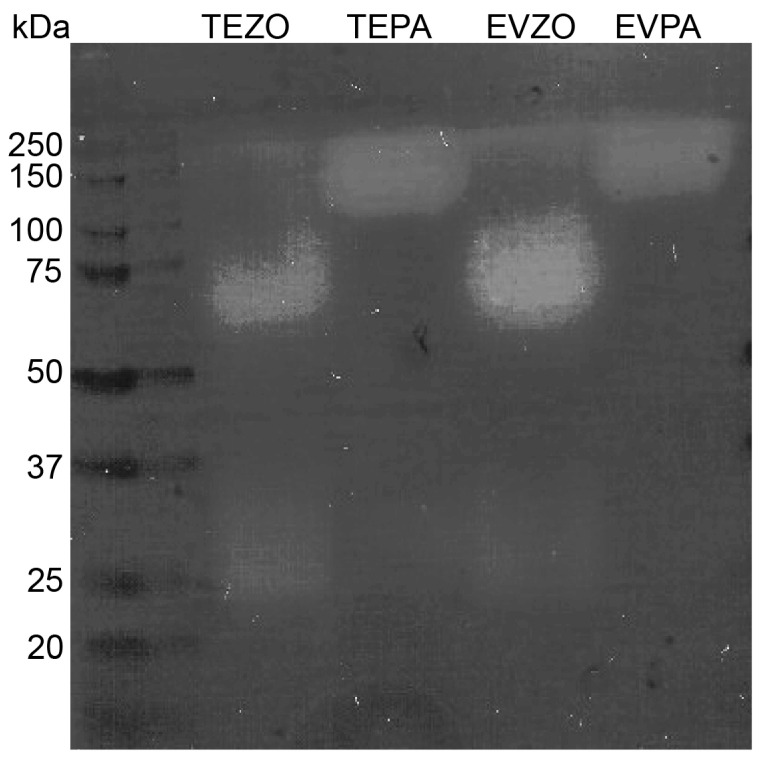
Zymography on polyacrylamide gel polymerized with 1% gelatin. Fifty micrograms of EV and TE were loaded in duplicate for ginger and papaya, and arrows indicate positive bands with proteolytic activity. EVZO: extracellular vesicles from *Zingiber officinale*; TEZO: total extract of *Zingiber officinale*, EVPA: extracellular vesicles from papaya, TEPA: total extract of papaya, kDa: kilodaltons.

Considering the results from the proteomics and GO analysis, EVZO contains proteins with proteolytic activity, and there was a decrease in collagen deposition in the BLM+EVZO group. We hypothesize that EVZO has the intrinsic ability to break down collagen in the ECM. Thus, we performed a zymography analysis in which we found an apparent pattern of proteolytic activity. A total of two bands were identified at approximate sizes of 75 and 30 kDa in TEZO and EVZO. In contrast, in our positive control, which was papaya, two bands were identified in the TE, one of 25 kDa and another with an approximate weight of 200 kDa, the latter being the most prominent, and EVPA showed only one band with proteolytic activity with a weight of 200 kDa. This result suggests that TEZO and EVZO have proteolytic activity (Figure 6).

### 3.6. EVZO Administration Decreases Myofibroblast Expression

**Figure 7:**
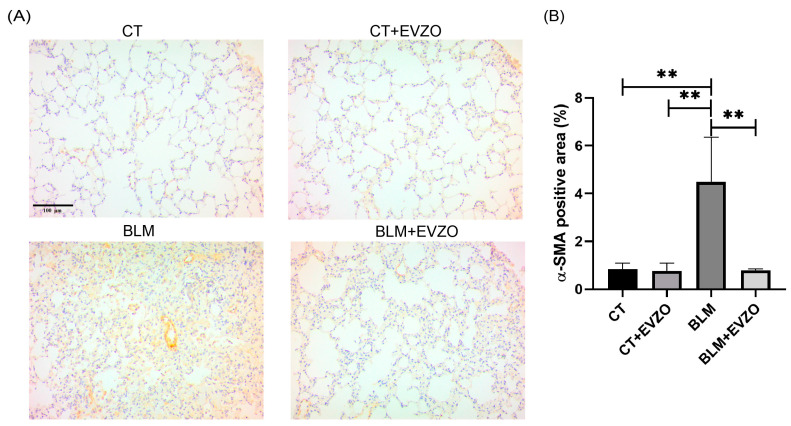
EVZO inhibits the expression of the myofibroblast marker α-SMA in cells. (**A**) Representative images were taken at 20× magnification of α-SMA expression and (**B**) quantification of positive areas. Data are expressed as the mean ± SD (*n* = 4). Quantification was performed with ImageJ 1.53t software and analyzed in GraphPad Prism. ** *p* < 0.01. BLM: bleomycin, CT: control, EVZO: extracellular vesicles from *Zingiber officinale*.

One of the main ECM-producing cells during IPF progression are myofibroblasts, which express α-SMA [31]. To evaluate the effects of EVZO on myofibroblasts, we performed immunohistochemistry of α-SMA. The analyses showed basal expression of α-SMA in the CT and CT+EVZO groups, while the BLM group showed a significant increase compared to controls. In contrast, the BLM+EVZO group showed a significant reduction in α-SMA expression compared to the BLM group (Figure 7A,B). This result suggests that treatment with EVZO decreases the expression of α-SMA-positive cells in the BLM-induced IPF model.

### 3.7. EVZO Modulates Inflammation and Lipid Peroxidation in IPF

**Figure 8:**
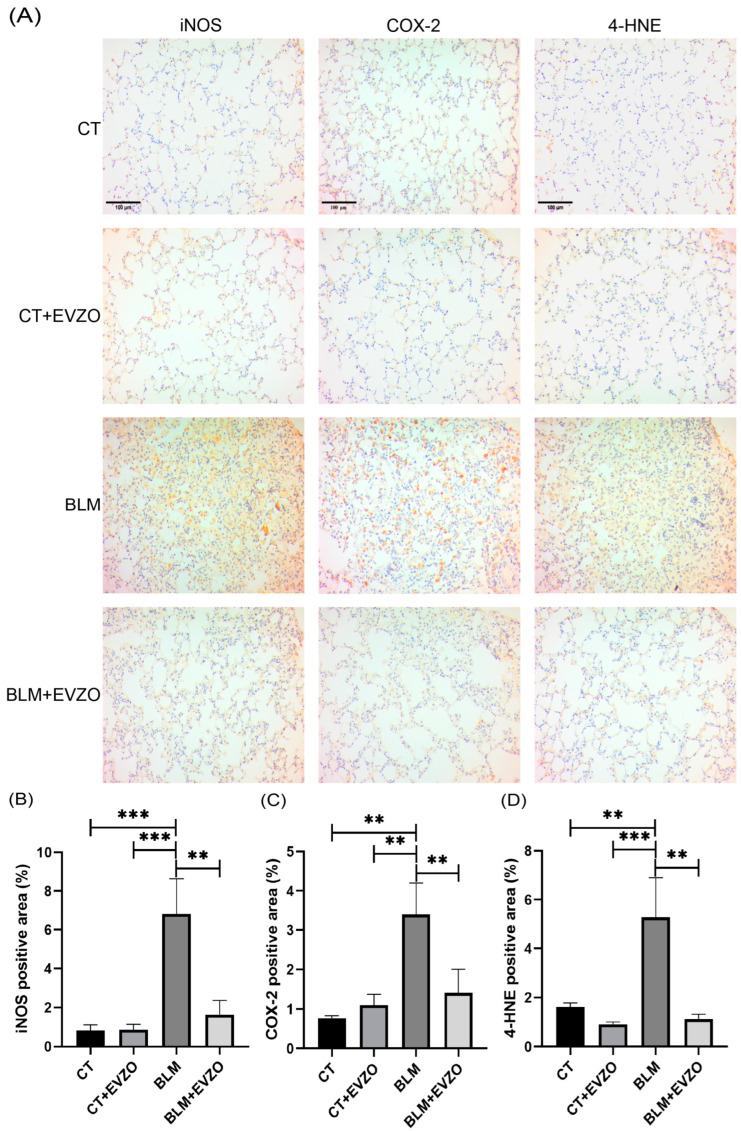
Modulation of inflammation and lipid peroxidation. (**A**) Representative images were taken at 20× magnification of iNOS, COX-2, and 4-HNE expression. (**B**) Quantification of positive areas of iNOS, (**C**) COX-2 and (**D**) 4-HNE expression. The results expressed are the mean ± SD (*n* = 4). Quantification was performed with ImageJ 1.53t software and analyzed in GraphPad Prism. ** *p* < 0.01, *** *p* < 0.001. BLM: bleomycin, CT: control, EVZO: extracellular vesicles *Zingiber officinale*, iNOS: inducible nitric oxide synthase, COX-2: cyclooxygenase-2, 4-HNE: 4-hydroxy-2-nonenal.

Inflammation plays an essential role in developing IPF and activating myofibroblasts. The secretion of nitric oxide (NO) by inducible nitric oxide synthase (iNOS) is involved in inflammation, respiratory processes of the lung parenchyma, and ECM remodeling (21). We evaluated the expression of iNOS by immunohistochemistry, and the analyses showed that the CT and CT+EVZO groups had basal expression. In contrast, the BLM group showed a significant increase compared to the control groups. The BLM+EVZO group significantly decreased the expression of iNOS compared to the BLM group (Figure 8A,B). Cyclooxygenases have also been associated with high expression in the pathogenesis of IPF, inflammation, and airway hyperreactivity. There are two COX isoforms, COX-1 and COX-2 [32,33]. We evaluated COX-2 expression by immunohistochemistry. The BLM group had significantly increased COX-2 expression compared to the CT and CT+EVZO groups, while the BLM+EVZO group showed a significant decrease compared to the BLM group and values close to those of the CT and CT+EVZO groups. (Figure 8A,C).

Moreover, the progression of IPF is favored by an oxidative environment, where an increase in reactive oxygen species (ROS) induces lipid peroxidation products such as 4-HNE that play a critical role in developing this pathology [34]. Therefore, to evaluate the effect of EVZO on 4-HNE expression in our model, we performed immunohistochemistry and observed that the CT and CT+EVZO groups had basal expression. In contrast, after BLM treatment, 4-HNE expression significantly increased compared to the respective controls, and the BLM+EVZO group showed a significant decrease similar to the CT and CT+EVZO groups (Figure 8A,C). Taken together, these results suggest that EVZO has an anti-inflammatory effect by regulating the expression of iNOS and COX-2, as well as a decrease in lipid peroxidation products induced in a BLM-induced IPF model.

### 3.8. EVZO SOD Activity

**Figure 9:**
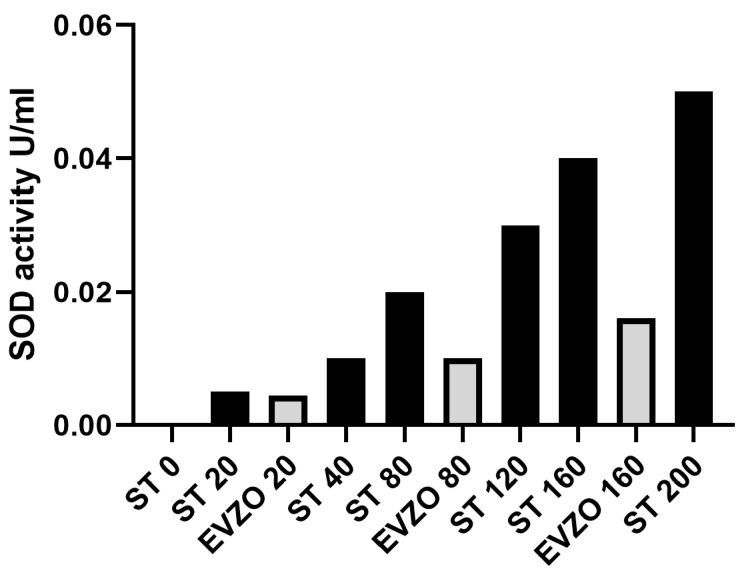
EVZO enzyme activity. SOD activity curve in standard and EVZO samples. ST: standard, EVZO: extracellular vesicles from *Zingiber officinale*.

The proteomic analysis showed the presence of proteins with antioxidant capacity, such as SOD, and the immunohistochemical analysis of 4-HNE showed that its expression decreased in the BLM+EVZO group, suggesting that EVZO possesses antioxidant activity. To confirm this, we evaluated their enzymatic activity through tetrazolium salt for the detection of superoxide radicals generated by xanthine oxidase and hypoxanthine. We observed that the higher the concentration of EVZO, the higher the SOD activity, suggesting that EVZO has functional SOD-antioxidant activity (Figure 9).

### 3.9. EVZO Decreases Apoptosis during the Progression of IPF

**Figure 10:**
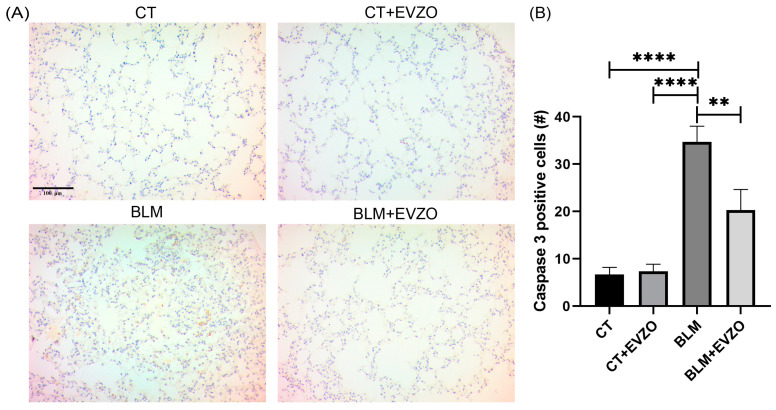
Expression of caspase 3, an indicator of apoptosis. (**A**) Representative images were taken at 20× magnification of tissue sections labeled with Caspase 3. (**B**) Quantification of Caspase 3 positive areas. The results are expressed as the mean ± SD *(n* = 4). Quantification was performed with ImageJ 1.53t software and analyzed in GraphPad Prism. ** *p* < 0.01, **** *p* < 0.0001. BLM: bleomycin, CT: control, EVZO: extracellular vesicles from *Zingiber officinale*.

Studies have reported that BLM increases ROS, causing oxidative stress and, consequently, mitochondrial damage and apoptosis [35,36]. In this study, to evaluate the effects of EVZO on BLM damage, immunohistochemistry was performed to measure the expression of apoptotic cells using the caspase-3 marker. We observed in the analysis that the CT and CT+EVZO groups expressed basal levels. In contrast, the BLM group showed a significant increase compared to the respective controls, and the BLM+EVZO group showed a significant decrease in the expression of caspase-3 compared to the BLM group (Figure 10A,B). This result suggests that EVZO can inhibit cell apoptosis in a BLM-induced IPF model.

## 4. Discussion

IPF is a chronic, progressive, fibrosing interstitial pneumonia of unknown etiology with a poor prognosis and currently has no effective pharmacological treatment [37]. For years, traditional medicine has been used to treat multiple diseases worldwide using plant extracts and derivatives, which are proposed as a complementary alternative to existing treatments [38]. Ju S. and collaborators showed that plants secrete their content in EVs, which are similar to those of mammals. These EVs can modulate a response by transferring their content to recipient cells such as stem cells, macrophages, and epithelial cells [22].

In this study, we evaluated the effect of EVZO in a BLM-induced IPF model. Our results showed that we isolated a population of EVZO with morphology and heterogeneous sizes similar to those obtained by Zhan et al. [13]. Furthermore, we demonstrated by label-free proteomic analysis a total of 20 proteins that, by GO analysis, participate in molecular functions, such as hydrolase, proteolysis, oxidoreductase, and antioxidant. Some proteins identified in this analysis are shared with those identified in the total extract in the study performed by Yin, Xiaojian, and collaborators [39,40].

It is important to mention that some of these identified proteins have been studied for their proteolytic activity; for example, the Zingipain protein is a cysteine proteinase with specific activity toward peptides with a proline residue in the P2 position. Due to its function as an endopeptidase, some authors have evaluated its function and found that it can hydrolyze collagen [41,42]. To verify that EVZO had this property, we performed a zymography assay, demonstrating for the first time that EVZO had intrinsic proteolysis activity. This effect in our study resembles the assay performed by Liu W and collaborators, who demonstrated that proteases obtained from ginger possess proteolytic activity when hydrolyzing fish skin gelatin [43].

Most drugs are administered orally; however, in this study, we administered EVZO via the nasal route, which offers advantages over other therapies, showing better bioavailability due to a rich vasculature and avoidance of hepatic elimination and targeted delivery to the lungs. Furthermore, they have shown that EVs are resistant to different media and enzymatic degradation, as evidenced by the administration of hydrochloride dihydrate in microspheres obtained from *Caesalpinia pulcherrima* pods and administered via the nasal route [44].

Our histological analyses revealed that EVZO treatments significantly decreased collagen deposits, which favored the recovery of the size of the alveolar spaces and decreased cellular infiltrate. Fibroblastic foci are associated with IPF progression and poor prognosis in fibrotic regions. These fibroblastic foci lead to the accumulation of myofibroblasts, the central cells that remodel the ECM [45,46]. Myofibroblasts are characterized by being positive for α-SMA, a marker shown to have elevated expression in murine models and human lung tissues with IPF. Our results showed that the group treated with BLM showed elevated expression. At the same time, EVZO treatment significantly decreased α-SMA expression, which is related to what was observed in Masson’s trichrome staining, which evidenced reduced collagen deposits. Interestingly, our results show no histological changes in the control group treated with EVZO (CT+EVZO), suggesting that it does not exert cytotoxic effects in our model, similar to in other cell types such as RAW 264.7 cells and colon epithelial cells that have also evaluated the cell viability of healthy cells when treated with ginger extract and EVs without showing toxic effects [13].

Total ginger extract has been shown to possess anti-inflammatory and antioxidant effects evaluated in vivo and in vitro by preventing the production of ROS and inflammatory markers such as iNOS and COX-2 [19,47]. Our study showed that BLM induced a significant increase in these inflammatory markers, while EVZO administration decreased their expression. In this BLM-induced IPF model, oxidative stress also plays an essential role in pathogenesis that can modulate the expression of profibrotic cytokines.

Damage caused by oxidative stress can increase the expression of profibrotic and inflammatory cytokines such as TGFβ, suggesting the presence of a vicious cycle; the most toxic form of oxidative stress is the expression of 4-HNE, a product of lipid peroxidation. [48,49]. Ganiyu Oboh et al. (2010) showed that ginger could decrease lipid peroxidation and therefore decrease oxidative stress and the secretion of profibrotic cytokines. Consistent with this, in our study, we observed that BLM significantly increased 4-HNE expression, but EVZO administration decreased its expression. These results suggest that EVZO has anti-inflammatory and antioxidant effects. To confirm the antioxidant effect of EVZO, we evaluated its enzymatic activity by increasing the production of CuZn-SOD, which is known to be the first line of defense in the antioxidant response by preventing free radical-induced damage. This effect was also observed in the study by Hua Li Y. et al. (2022), who determined the activity of SOD in total ginger extract and observed an increase in SOD production [50]. Another study evaluated patients who received cycles of chemotherapy and were treated with ginger and found increased oxidative defense by increasing CuZn-SOD, GPx, and GSH/GSSG [51].

Apoptosis is a natural mechanism by which cells induce programmed cell death and is a consequence of ROS produced in damaged cells [52]. In our fibrosis model, BLM generated a significant increase in apoptotic cells, as assessed by caspase-3 expression. This result agrees with studies previously reported by Mungunsukh O et al. (2010) in their model of BLM-induced lung damage, where an increase in apoptotic cells and histological alterations generated by an oxidative stress environment were induced [36]. However, in our study, the damage was abolished with EVZO administration, suggesting that EVZO exerts an anti-apoptotic effect in BLM-damaged tissues by decreasing cell death. This result resembles that achieved by Baiomy et al., who evaluated that cadmium toxicity caused liver and kidney damage with ROS production, which led cells to apoptosis. Nevertheless, after treatment with ginger extract, there was a decrease in caspase-3-positive cells [53].

## 5. Conclusions

In the present study, we found that ginger-derived extracellular vesicles carry functional proteins with protease and SOD activity, suggesting that they help to decrease the damage caused by BLM in the mouse model, where they decreased the expression of markers of fibrogenesis, such as markers of oxidative stress, α-SMA collagen deposition, markers of inflammation, and cell apoptosis. This finding opens possibilities to develop new therapeutic strategies based on using natural product EVs carrying bioactive molecules. Despite these results, further studies are needed to evaluate their effects on other organs in this model of IPF.

## Figures and Tables

**Table 1:** Antibodies used in immunohistochemical staining.

Antibody	Dilution	Trading House
α-SMA (Smooth muscle actin alpha)	1:1000	Proteintech 55135-1-AP
iNOS (inducible nitric oxide synthase)	1:400	Abcam ab3523
COX-2 (cyclooxygenase-2)	1:800	Abcam ab15191
4-HNE (4-hydroxy-2-nonenal)	1:250	Abcam ab46545
Caspase-3	1:500	Cell Signaling 9662
Anti-rabbit secondary antibody	1:300	Abcam ab6721

**Table 2:** Proteins identified in ginger-derived EVs. Two independent EVZO samples were analyzed.

N.	ID	Protein Name
1	A0A088QCR4	ATP synthase subunit beta
2	A0A0B4UM59	NADPH-dependent double-bond reductase 2
3	A0A3G2Z6Y7	ATP synthase CF0 subunit I
4	A0A5C0F4T1	Ribulose bisphosphate carboxylase large chain
5	A0A3G2Z7J8	ATP-dependent Clp protease proteolytic subunit
6	A0A5C0F797	Protein TIC 214
7	A0A8F6UC51	Caffeoyl-coenzyme A O-methyltransferase
8	B3FYN2	30S ribosomal protein S19, chloroplastic
9	Q3L634	Mannose-binding lectin
10	C0HK70	Superoxide dismutase [Cu-Zn]
11	E3WEA5	NADPH--cytochrome P450 reductase
12	E3WEA6	NADPH--cytochrome P450 reductase
13	H9BXB8	Cytosolic glyceraldehyde-3-phosphate dehydrogenase
14	P82473	Zingipain-1
15	P82474	Zingipain-2
16	Q1ZZ93	Chlorophyll a-b binding protein, chloroplastic
17	Q5ILG4	Cysteine protease gp3b
18	Q5ILG5	Cysteine protease gp3a
19	Q5ILG6	Cysteine protease gp2b
20	Q5ILG7	Cysteine protease gp2a

## Data Availability

Not applicable.
